# A single arm Phase I/II trial on the combination of carboplatin, nab-paclitaxel and avastin as first-line treatment for advanced non-squamous non-small cell lung cancer (TORG1424/OLCSG1402: CARNAVAL)

**DOI:** 10.1093/jjco/hyae044

**Published:** 2024-04-09

**Authors:** Naoyuki Nogami, Toshio Kubo, Akihiro Bessho, Makoto Sakugawa, Satoshi Ikeo, Toshihide Yokoyama, Nobuhiko Seki, Ryosuke Ochiai, Nobukazu Fujimoto, Shuji Murakami, Kyoichi Kaira, Toshiyuki Harada, Daizo Kishino, Yuichi Takiguchi, Tsuneo Shimokawa, Katsuyuki Kiura, Natsumi Yamashita, Hiroaki Okamoto

**Affiliations:** Department of Community Medicine, Pulmonology and Cardiology, Ehime University Graduate School of Medicine, Toon, Japan; Center for Clinical Oncology, Okayama University Hospital, Okayama, Japan; Department of Respiratory Medicine, Japanese Red Cross Okayama Hospital, Okayama, Japan; Department of Respiratory Medicine, Japanese Red Cross Okayama Hospital, Okayama, Japan; Department of Respiratory Medicine, Kurashiki Central Hospital, Kurashiki, Japan; Department of Respiratory Medicine, Kurashiki Central Hospital, Kurashiki, Japan; Division of Medical Oncology, Department of Internal Medicine, Teikyo University School of Medicine, Tokyo, Japan; Division of Medical Oncology, Department of Internal Medicine, Teikyo University School of Medicine, Tokyo, Japan; Department of Respiratory Medicine, Okayama Rosai Hospital, Okayama, Japan; Department of Thoracic Oncology, Kanagawa Cancer Center, Yokohama, Japan; Department of Allergy and Respiratory Medicine, Gunma University Graduate School of Medicine, Maebashi, Japan; Department of Respiratory Medicine, Japan Community Health Care Organization Hokkaido Hospital, Sapporo, Japan; Department of Respiratory Medicine, Japanese Red Cross Himeji Hospital, Himeji, Japan; Department of Medical Oncology, Graduate School of Medicine, Chiba University, Chiba, Japan; Department of Respirology Medicine and Medical Oncology, Yokohama Municipal Citizen’s Hospital, Yokohama, Japan; Department of Allergy and Respiratory Medicine, Okayama University Hospital, Okayama, Japan; Clinical Research Center, National Hospital Organization Shikoku Cancer Center, Matsuyama, Japan; Department of Respirology Medicine and Medical Oncology, Yokohama Municipal Citizen’s Hospital, Yokohama, Japan

**Keywords:** NSCLC, Japanese population, neutropenia, dosing scheme, bevacizumab

## Abstract

**Background:**

Bevacizumab with platinum doublet therapy including paclitaxel + carboplatin improves the survival of patients with non-squamous non-small cell lung cancer. However, in a previous trial (CA031), paclitaxel + carboplatin led to Grade > 3 neutropenia in a Japanese population. Nanoparticle albumin-bound paclitaxel exhibits an improved toxicity profile. We evaluated the safety, dosage and response rate of the nanoparticle albumin-bound paclitaxel + carboplatin + bevacizumab combination in a Japanese population.

**Methods:**

Chemotherapy-naive patients with advanced non-squamous non-small cell lung cancer were included. The dosage schedule was established in the Phase I trial as follows: 4–6 cycles of carboplatin (area under the concentration–time curve = 6 on Day 1) + nanoparticle albumin-bound paclitaxel (100 mg/m^2^ on Days 1, 8 and 15) + bevacizumab (15 mg/kg on Day 1), followed by maintenance therapy (nanoparticle albumin-bound paclitaxel + bevacizumab). The response rate and presence of adverse effects were evaluated in the Phase II trial.

**Results:**

The overall response rate was 56.5% (90% confidence interval: 44.5–68.5), and 93% of patients (43/46) showed tumor shrinkage or maintained a stable disease course. The primary endpoint was achieved. At the median follow-up duration of 42 months, the median overall survival was 18.9 (range: 10.5–32.4) months. The most frequently observed Grade ≥ 3 adverse effects were neutropenia (72%), leukopenia (50%) and anemia (30%).

**Conclusions:**

All adverse effects were manageable and none resulted in patient death. In conclusion, the nanoparticle albumin-bound paclitaxel + carboplatin + bevacizumab combination is favorable and well tolerated in Japanese patients as first-line treatment for advanced non-squamous non-small cell lung cancer.

## Teaser

The combination of the nanoparticle albumin-bound paclitaxel + carboplatin + bevacizumab as first-line treatment for advanced non-squamous non-small cell lung cancer is revealed to be favorable and well tolerated in Japanese patients.

## Introduction

Lung cancer is the leading cause of mortality worldwide (1). Non-small cell lung cancer (NSCLC) accounts for 85% of all lung cancers and can be subdivided into squamous and non-squamous cell carcinomas, including adenocarcinoma and large-cell carcinoma. More than half of the patients with NSCLC are diagnosed with advanced disease and receive chemotherapy, molecular targeted therapy or immunotherapy. In patients with advanced NSCLC, platinum doublet chemotherapy leads to prolonged survival compared with the best supportive care (2–6). However, the prognosis for these patients remains poor, with a 1-year survival rate of 30–40% reported in patients treated with chemotherapy (7); therefore, advances in treatment regimens are needed.

Bevacizumab (BEV) is a monoclonal antibody-targeting vascular endothelial growth factor that normalizes vascular permeability, which then increases the intratumoral concentration of paclitaxel (PTX) (8). The ECOG4599 trial that compared the effect of carboplatin (CBDCA) + PTX therapy with or without BEV reported a prolongation of overall survival (OS) (10.3 vs. 12.3 months, hazard ratio [HR] = 0.79, 95% confidence interval [CI]: 0.67–0.92) and progression-free survival (PFS) (4.5 vs. 6.2 months, HR = 0.66, 95% CI: 0.57–0.77) with the use of BEV (9). In the AVAPERL Phase III study, BEV + pemetrexed (PEM) combination therapy for maintenance extended the PFS compared with BEV alone (7.4 vs. 3.7 months), and was well tolerated (10,11). Although the incidence of adverse effects (AEs) was high in the BEV + PEM arm, the health-related quality of life was comparable with the improved efficacy of treatment (12). However, the Japanese randomized Phase II JO19907 trial that compared the positive effects of BEV and CBDCA + PTX reported no significant difference in OS, but confirmed prolonged PFS (13).

Nanoparticle albumin-bound paclitaxel (nab-PTX) is a nanoparticle human serum albumin-bound paclitaxel that can overcome some issues associated with paclitaxel, such as hypersensitivity to solvents or alcohol (14). In an international Phase III trial comparing combination treatment with PTX + CBDCA (PTX arm) and nab-PTX + CBDCA (nab-PTX arm) (CA031 trial), the primary response rate was 25% in the PTX arm and 33% in the nab-PTX arm, with the nab-PTX arm showing a significant advantage over the PTX arm (15). Interestingly, the percentage of patients with AEs, specifically Grade ≥ 3 neutropenia, was higher in the Japanese population (16).

A Phase II trial in patients with advanced non-squamous NSCLC evaluated the efficacy of combination therapy with nab-PTX + CBDCA + BEV as first-line treatment; the response rate was 36% (13/36) and the median PFS and OS were 8.5 and 12.2 months, respectively. The study treatment was discontinued in five patients due to AEs (17). However, it remains unclear whether combination therapy with nab-PTX + CBDCA + BEV is effective and tolerable as first-line treatment in Japanese patients with non-squamous NSCLC.

The objective of this trial was to assess the efficacy and safety of the combination of CBDCA + BEV + nab-PTX as first-line treatment in chemotherapy-naïve Japanese patients with stage IIIB/IV non-squamous NSCLC. The Phase I trial was designed to evaluate the safety and recommended dosage (RD) in this population, which was then used in the Phase II trial, in which the primary objective was to evaluate the overall response rate. The secondary endpoints included OS, PFS and toxicity.

## Patients and Methods

### Study design

This clinical trial was an open-label, single-arm prospective Phase I/II trial designed in accordance with the Declaration of Helsinki and the Ethical Guidelines for Clinical Research issued by the Japanese Ministry of Health, Labour, and Welfare. The protocol was approved by the institutional review boards of all the participating institutions. The clinical trial registry number is UMIN000014560. The following hospitals participated in the study: Okayama University Hospital, National Hospital Organization Shikoku Cancer Center, Japanese Red Cross Okayama Hospital, Kurashiki Central Hospital, Okayama Rosai Hospital, Kanagawa Cancer Center, Japan Community Health Care Organization Hokkaido Hospital, Japanese Red Cross Himeji Hospital, Teikyo University Hospital, Gunma University Hospital, Chiba University Hospital and Yokohama Municipal Citizen’s Hospital.

### Eligibility criteria

**Figure 1:**
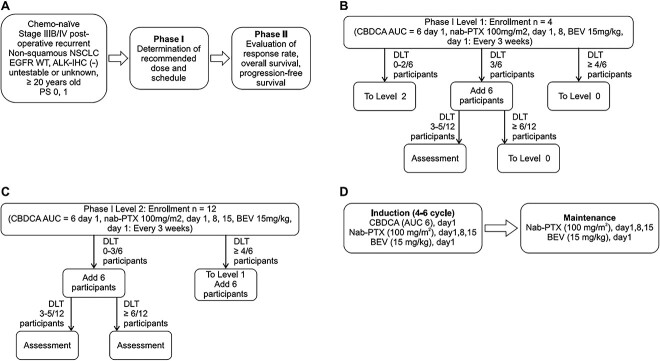
Overview of the clinical trial design. (A) Selection criteria; (B) Phase I, dosage Level 1; (C) Phase I, dosage Level 2; (D) Phase II, dosage.

Eligible patients were ≥20 years old with confirmed non-squamous stage IIIB/IV NSCLC, who could not be treated with surgical operation or definitive chemoradiotherapy, were chemotherapy-naïve, had a quantifiable disease with an Eastern Cooperative Oncology Group (ECOG) performance status (PS) of 0 or 1, and had satisfactory hematologic, hepatic and renal functions. Patients were excluded if they had symptomatic brain metastasis, a history of hemoptysis, uncontrolled hypertension, gastrointestinal hemorrhage, perforation, fistula, diverticulitis, Grade ≥ 2 neuropathy, a history of allergy or hypersensitivity to the study drugs or albumin, concomitant malignancies, pleural effusion, ascites, or pericardial effusion requiring treatment such as drainage or were taking anticoagulants (aspirin in doses ≤325 mg was allowed). Written informed consent was obtained from all patients. The clinical trial design is summarized in Figure 1A.

### Criteria for treatment

The major criteria for Day 1 treatment were a neutrophil count ≥1500/mm^3^, platelet count ≥100 000/mm^3^ and neuropathy ≤ Grade 1; those on Days 8 and 15 were a neutrophil count ≥1000/mm^3^, platelet count ≥50 000/mm^3^ and neuropathy ≤ Grade 1.

### Dose and dosage in Phase I

In Phase I, patients received 4–6 cycles of CBDCA (dosage Level 0, area under the concentration–time curve [AUC] = 5 on Day 1; dosage Levels 1 and 2, AUC = 6 on Day 1) + nab-PTX (dosage Levels 0 and 1, 100 mg/m^2^ on Days 1 and 8 or dosage Level 2, 100 mg/m^2^ on Days 1, 8 and 15) + BEV (15 mg/kg on Day 1), followed by maintenance therapy with nab-PTX + BEV every 3 weeks until the disease progressed, an unacceptable toxicity developed or patients requested to discontinue the protocol treatment. The RD and dosage schedule in Cycle 1 in Phase II were based on dose-limiting toxicity (DLT), which occurred until the initiation of Cycle 2. DLTs were defined as: Grade 4 neutropenia that continued for >4 days; febrile neutropenia; Grade 4 thrombocytopenia; non-hematologic toxicity of Grade 3 or higher, excluding nausea, vomiting, anorexia, fatigue, diarrhea, constipation, electrolyte abnormalities, hypertension and hypersensitivity reactions, which can be managed with appropriate treatment; Grade 4 hypertension; initiation of cycle 2 delayed for >14 days from the planned treatment day due to AEs; and unplanned interruption of treatment on Day 8 at Levels 1 and 0 or on both Days 8 and 15 at Level 2 due to AEs. The trial started from Level 1. It followed a 6 + 6 escalation design, in which six patients were enrolled in Level 1, and if the predefined DLT was observed in ≤2 patients, six more patients were enrolled in Level 2. If the predefined DLT at Level 1 occurred in three patients, additional patients were enrolled in Level 1 to evaluate the toxicity. If the predefined DLT at Level 1 occurred in ≥4 patients, six patients were enrolled in Level 0. If six additional patients were enrolled in Level 1, and DLT occurred in 3–5 patients out of 12, the trial was continued with the evaluation of the RD and dosage schedule. However, if DLT was observed in ≥6 of 12 patients, the patients were enrolled in Level 0. The 6 + 6 escalation design for Level 1 is summarized in Figure 1B and Level 2 in Figure 1C.

### Dosage and dosage schedule in Phase II

The RD and dosage schedule were determined in the Phase I trial as follows: 4–6 cycles of CBDCA (AUC = 6 on Day 1) + nab-PTX (100 mg/m^2^ on Days 1, 8 and 15) + BEV (15 mg/kg on day 1), followed by maintenance therapy (nab-PTX + BEV) every 3 weeks (Fig. 1D) until the patient met the criteria for discontinuation of protocol treatment (pneumonitis of ≥Grade 2, non-hematologic toxicity of ≥Grade 4, protocol treatment was judged to be ineffective or initiation of another anticancer treatment was deemed necessary; criteria for starting treatment were not met by Day 43 from Day 1 of the previous cycle, death, patient was found to be ineligible or transferred to another hospital, patient requested discontinuation of the protocol due to AEs or reasons unrelated to AEs, patient withdrew consent, or physician/investigator determined that the protocol treatment needed to be discontinued).

### Toxicity and response assessment

Toxicities were evaluated according to the National Cancer Institute Common Terminology Criteria for AEs version 4.0. Tumor response was assessed using the Response Evaluation Criteria in Solid Tumors (RECIST), version 1.1.

### Statistical analysis

The primary objectives were to determine the RD and dosage schedule of CBDCA + nab-PTX + BEV treatment in Phase I and to evaluate the overall response rate in Phase II. In Phase I, treatment was administered to 6–12 patients at each dose level; therefore, the number of patients that needed to be enrolled in Phase I was 12–24. According to previous studies, the threshold of overall response rate of combination therapy is 30%, and the expected response rate was 50% (9,13,16). Calculation of the necessary number of subjects using *α* = 0.05 (one-sided) and *β* = 0.1 yielded 49 patients; considering that some patients will be ineligible, the planned sample size for Phase II was set at 55. PFS was defined as the duration from the date of enrolment to the diagnosis of progression or death from any cause, whichever occurred sooner. OS was defined as the duration from the date of enrolment to death from any cause. A full analysis set was used for statistical analysis. PFS and OS curves were estimated using the Kaplan–Meier method; median PFS, OS and 95% two-sided CIs were calculated using the Brookmeyer and Crowley method (18). An interim analysis of the primary endpoint was not performed in this study.

## Results

### Baseline patient characteristics in Phases I and II

Phase I lasted from October 2014 to July 2015; 4 and 12 patients were enrolled in the dosage Levels 1 and 2, respectively. Overall, the median age was 65 years, and 11 (68.8%) patients were male. Most patients exhibited an ECOG PS of 1 (75.0%) and had adenocarcinoma (81.2%). All patients were in Stage IV of the disease and epidermal growth factor receptor (EGFR) mutation-negative. In Phase II, the enrolment continued for 4 years, from November 2015 to July 2018, and 46 patients were enrolled. The median follow-up period for surviving patients was 18.2 months. The median age was 66 (45–79) years, and 35 (76.1%) patients were male. Most patients were in Stage IV (78.3%), had adenocarcinoma (89.1%) and were EGFR mutation-negative (76.1%) and ALK fusion-negative (89.1%) (Table 1).

**Table 1:** Baseline patient characteristics

	Phase I Level 1 *n* = 4	Phase I Level 2 *n* = 12	Phase II *n* = 46
Gender Male Female	3 (75%) 1 (25%)	8 (66.7%) 4 (33.3%)	35 (76.1%) 11 (23.9%)
Age (years) Median (range)	66 (65–69)	63 (52–71)	66 (45–79)
Histological type Adenocarcinoma Others (NOS)	4 (100%) 0 (0%)	9 (75.0%) 3 (25.0%)	41 (89.1%) 5 (10.9%)
Stage Postoperative recurrence Stage III B Stage IV	0 (0%) 0 (0%) 4 (100%)	0 (0%) 0 (0%) 12 (100%)	8 (17.4%) 2 (4.3%) 36 (78.3%)
ECOG PS at initiation of therapy 0 1	1 (25%) 3 (75%)	3 (25%) 9 (75%)	13 (28.3%) 33 (71.7%)
Smoking history No Yes	1 (25%) 3 (75%)	4 (33.3%) 8 (66.7%)	12 (26.1%) 34 (73.9%)
EGFR gene mutation Positive Negative Undetectable/unknown	0 (0%) 4 (100%) 0 (0%)	0 (0%) 12 (100%) 0 (0%)	10 (21.7%) 35 (76.1%) 1 (2.2%)
ALK gene mutation Positive Negative Undetectable/unknown	0 (0%) 4 (100%) 0 (0%)	0 (0%) 11 (91.7%) 1 (8.3%)	0 (0%) 41 (89.1%) 5 (10.9%)
History of TKI treatment Yes No	0 (0%) 4 (100%)	0 (0%) 12 (100%)	11 (23.9%) 35 (76.1%)

NOS: not otherwise specified; ECOG PS: Eastern Cooperative Oncology Group performance status; TKI: tyrosine kinase inhibitor; NA: not applicable.

### Treatment course and observed AEs in Phase I

Four patients without DLT were treated at dosage Level 1. We decided to proceed to Level 2 as predefined DLTs occurred in ≤2 patients. Twelve patients without DLT were treated at dosage Level 2, and the RD in Phase II was considered as dosage Level 2. Although 1 patient at dosage Level 1 experienced Grade ≥ 3 nausea and vomiting before the administration of nab-PTX on Day 8, which was managed with appropriate treatment, it was not considered as DLT because the patient requested discontinuation of the protocol treatment (Table 2). The median number of treatment cycles was 8 (range, 1–24). Grade ≥ 3 AEs at dosage Level 1 were as follows: neutropenia (75.0%), thrombocytopenia (50.0%), leukopenia, nausea, vomiting, liver disorder and skin ulceration (25.0% each). At dosage Level 2, the following Grade ≥ 3 AEs occurred: neutropenia (83.3%), leukopenia (58.3%), loss of appetite (25.0%), thrombocytopenia, anemia, nausea, vomiting, fatigue, hypertension (16.7% each), hyponatremia, pneumonitis, febrile neutropenia, esophageal perforation and edema (8.3% each). No treatment-related deaths were observed (Table 3). Although two patients decided to discontinue owing to AEs (nausea, vomiting), all other AEs were manageable.

**Table 2:** Summary of treatment course of Phase I

	Level 1 *n* = 4	Level 2 *n* = 12
Treatment course completed	2	7
Early discontinuation	2	5
Reason for discontinuation		
Adverse event	1^a^	2^a^
Patient wished to discontinue for reasons unrelated to adverse events	1	2
Patient wished to discontinue for reasons related to adverse events	0	1^b^
Patient died during treatment period	0	0

^a^Grade 3 pneumonitis, Grade 3 edema, unrecovered anal fistula.

^b^Nausea, vomiting.

**Table 3:** Adverse events observed in Phase I study

	Level 1 *n* = 4		Level 2 *n* = 12	
	Any (%)	≥G3 (%)	Any (%)	≥G3 (%)
Neutropenia	3 (75.0)	3 (75.0)	12 (100.0)	10 (83.3)
Leukopenia	3 (75.0)	1 (25.0)	11 (91.7)	7 (58.3)
Thrombocytopenia	3 (75.0)	2 (50.0)	9 (75.0)	2 (16.7)
Anemia	3 (75.0)	0 (0.0)	12 (100.0)	2 (16.7)
Nausea	2 (50.0)	1 (25.0)	6 (50.0)	2 (16.7)
Vomiting	1 (25.0)	1 (25.0)	4 (33.3)	2 (16.7)
Increased bilirubin	3 (75.0)	1 (25.0)	5 (41.7)	0 (0.0)
Increased AST	1 (25.0)	1 (25.0)	6 (50.0)	0 (0.0)
Increased ALT	1 (25.0)	1 (25.0)	8 (66.7)	0 (0.0)
Hyponatremia	2 (50.0)	0 (0.0)	6 (50.0)	1 (8.3)
Pneumonitis	1 (25.0)	0 (0.0)	1 (8.3)	1 (8.3)
Febrile neutropenia	0 (0.0)	0 (0.0)	1 (8.3)	1 (8.3)
Loss of appetite	2 (50.0)	0 (0.0)	8 (66.7)	3 (25.0)
Fatigue	4 (100.0)	0 (0.0)	9 (75.0)	2 (16.7)
Hypertension	1 (25.0)	0 (0.0)	3 (25.0)	2 (16.7)
Esophageal perforation	0 (0.0)	0 (0.0)	1 (8.3)	1 (8.3)
Edema of extremities	0 (0.0)	0 (0.0)	1 (8.3)	1 (8.3)
Edema of the body trunk	0 (0.0)	0 (0.0)	1 (8.3)	1 (8.3)
Increased GGT	1 (25.0)	1 (25.0)	1 (8.3)	0 (0.0)
Liver infection	1 (25.0)	1 (25.0)	0 (0.0)	0 (0.0)
Skin ulceration	1 (25.0)	1 (25.0)	0 (0.0)	0 (0.0)
Increased creatinine	2 (50.0)	0 (0.0)	5 (41.7)	0 (0.0)
Hypoalbuminemia	2 (50.0)	0 (0.0)	8 (66.7)	0 (0.0)
Increased ALP	2 (50.0)	0 (0.0)	4 (33.3)	0 (0.0)
Fever	2 (50.0)	0 (0.0)	1 (8.3)	0 (0.0)
Constipation	3 (75.0)	0 (0.0)	9 (75.0)	0 (0.0)
Diarrhea	0 (0.0)	0 (0.0)	5 (41.7)	0 (0.0)
Alopecia	2 (50.0)	0 (0.0)	8 (66.7)	0 (0.0)
Peripheral sensory neuropathy	2 (50.0)	0 (0.0)	8 (66.7)	0 (0.0)
Oral mucositis	3 (75.0)	0 (0.0)	5 (41.7)	0 (0.0)
Taste abnormality	0 (0.0)	0 (0.0)	7 (58.3)	0 (0.0)
Epistaxis	2 (50.0)	0 (0.0)	6 (50.0)	0 (0.0)

AST, aspartate aminotransferase; ALT, alanine aminotransferase; GGT, γ-glutamyl transpeptidase; ALP, alkaline phosphatase.

### Treatment course and response rate in Phase II

**Figure 2:**
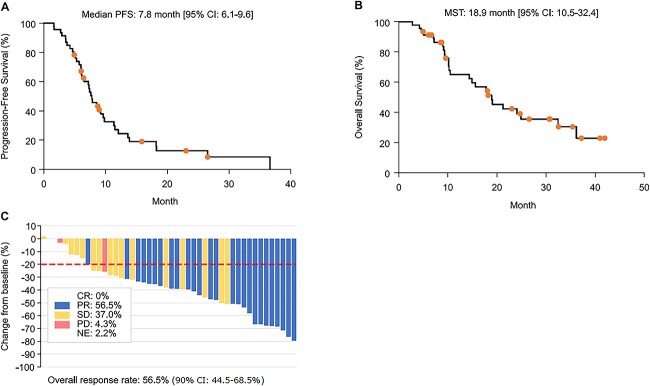
Therapeutic effect of nab-PTX + CBDCA + BEV. (A) PFS, progression-free survival. (B) OS, overall survival. (C) Waterfall plot of overall response rate, CR, complete response; PR, partial response; SD, stable disease; PD, progressive disease; NE, not evaluable; CI, confidence interval; nab-PTX, nanoparticle albumin-bound paclitaxel; CBDCA, carboplatin; BEV; bevacizumab.

The median number of treatment cycles was 7 (range, 1–31), with 27 patients (58.7%) receiving 4–6 cycles of induction treatment during the trial. After the treatment period, 58.7% (27/46) patients transitioned to maintenance therapy with nab-PTX + BEV. Four patients discontinued treatment at the end of the follow-up period, and 42 discontinued therapy due to AEs, disease progression or personal preference (Table 4). At the median follow-up duration of 36 months, the median PFS was 7.8 (range, 6.1–9.6) months (Fig. 2A), and at the median follow-up duration of 42 months, the median OS was 18.9 (range, 10.5–32.4) months (Fig. 2B). The overall response rate was 56.5% (90% CI: 44.5–68.5) (Fig. 2C), and the primary endpoint was achieved. Tumor shrinkage was evaluated according to RECIST 1.1. Although no patient achieved a complete response, 43 of 46 patients (93%) showed tumor shrinkage or maintained a stable disease course (Fig. 2C). Of the 46 patients in Phase II, 71.7% of patients required dose delay, 71.7% required dose reduction and 80.4% required drug discontinuation. Adverse events that led to discontinuation were pneumonitis (Grade 3: one patient, Grade 2: one patient), hyponatremia (Grade 4: one patient), infection (Grade 3: one patient), edema (Grade 3: one patient), atrial fibrillation (Grade 2: one patient), prolonged adverse events exceeding 21 days (peripheral sensory neuropathy Grade 2: four patients) and three-step dose reduction (pulmonary infection Grade 3: one patient, neutropenia, Grade 3: two patients). The following Grade ≥ 3 AEs were reported: neutropenia (71.7%), leukopenia (50%), anemia (30.4%), loss of appetite (13%), thrombocytopenia (10.9%), hyponatremia, hypertension, nausea (6.5% each), vomiting, increased aspartate aminotransferase level, fatigue (4.3% each), hypoalbuminemia, constipation, proteinuria, pneumonitis, esophageal perforation and febrile neutropenia (2.2% each) (Table 5). No treatment-related deaths were observed.

**Table 4:** Summary of treatment course of Phase II

	*n* = 46
Protocol treatment discontinued at the end of follow-up period	4
Early discontinuation reasons for discontinuation	42
Disease progression	18
Adverse events	13
Patient wanted to discontinue for reasons unrelated to adverse events	5
Patient wanted to discontinue for reasons related to adverse events	6
Death during treatment period	0

**Table 5:** Adverse events observed in Phase II study

	Any (%)	≥G3 (%)
Neutropenia	42 (91.3)	33 (71.7)
Leukopenia	43 (93.5)	23 (50.0)
Anemia	46 (100.0)	14 (30.4)
Loss of appetite	25 (54.3)	6 (13.0)
Thrombocytopenia	34 (73.9)	5 (10.9)
Hyponatremia	26 (56.5)	3 (6.5)
Hypertension	11 (23.9)	3 (6.5)
Nausea	22 (47.9)	3 (6.5)
Vomiting	10 (21.7)	2 (4.3)
Increased AST	21 (45.7)	2 (4.3)
Fatigue	20 (43.5)	2 (4.3)
Hypoalbuminemia	37 (80.4)	1 (2.2)
Constipation	28 (60.9)	1 (2.2)
Proteinuria	7 (15.2)	1 (2.2)
Pneumonitis	2 (4.3)	1 (2.2)
Esophageal perforation	1 (2.2)	1 (2.2)
Febrile neutropenia	1 (2.2)	1 (2.2)
Increased ALT	21 (45.7)	0 (0.0)
Creatinine increased	19 (41.3)	0 (0.0)
Diarrhea	14 (30.4)	0 (0.0)
Peripheral sensory neuropathy	23 (50.0)	0 (0.0)
Peripheral motor neuropathy	4 (8.7)	0 (0.0)
Alopecia	25 (54.3)	0 (0.0)
Thromboembolic event	4 (8.7)	0 (0.0)
Epistaxis	18 (39.1)	0 (0.0)

## Discussion

Previous studies showed that the administration of PTX + CBDCA resulted in higher hematological toxicity in Japanese patients than in American patients, despite the same dosage and dosage schedule (neutropenia: 70 vs. 38%, febrile neutropenia: 12 vs. 2%) (19). Similarly, the CA031 trial demonstrated that the combination of nab-PTX + CBDCA resulted in a higher incidence of Grade ≥ 3 neutropenia in Japanese patients than in the entire population (69 vs. 47%) (16). Therefore, we first evaluated the RD of combination therapy with nab-PTX + CBDCA + BEV as first-line therapy for Japanese patients with non-squamous NSCLC. Four patients were treated at dosage Level 1 (4–6 cycles of CBDCA, AUC = 6 on Day 1, nab-PTX 100 mg/m^2^ on Days 1 and 8, and BEV 15 mg/kg on Day 1), and 12 at dosage Level 2 (4–6 cycles of CBDCA, AUC = 6 on Day 1, nab-PTX 100 mg/m^2^ on Days 1, 8 and 15, and BEV 15 mg/kg on Day 1). No DLT was observed; therefore, dosage Level 2 was used to evaluate treatment response. However, dose delay or reduction was required in most patients at this level, similar to that in the CA031 trial, in which 82% of the patients required dose delay and 46% required dose reduction (16). Therefore, dose modifications for the therapeutic regimen used in this study were required.

The primary endpoint for Phase II was the response rate. According to the results of the CA031 study in patients with NSCLC, the response rate in the nab-PTX + CBDCA group based on histological findings was 33% for all histological types, 26% for adenocarcinoma and 33% for large-cell carcinoma (16). In addition, according to the results of the JO19907 trial involving patients with non-squamous NSCLC, the response rate was 31% (95% CI: 19.5–44.5) in the PTX + CBDCA group and 60.7% (95% CI: 51.2–69.6) in the PTX + CBDCA + BEV group ( 13). Furthermore, according to the results of the overseas E4599 study, the response rate was 15% in the PTX + CBDCA group and 35% in the PTX + CBDCA + BEV group ( 9). The response rate in this study was 56.5% (90% CI: 44.5–68.5, 95% CI: 42.2–70.8), which was greater than that reported in a previously mentioned study in a non-Japanese population (17). The Japanese randomized Phase II JO19907 trial that assessed the added benefit of BEV with CBDCA + PTX reported no significant difference in OS (22.8 vs. 23.4 months, HR = 0.99, 95% CI: 0.65–1.50), but confirmed prolonged PFS (5.9 vs. 6.9 months, HR = 0.61, 95% CI: 0.42–0.89) (13). In this study, the median PFS was 7.79 (6.08–9.59) months, and the median OS was 18.9 (range, 10.5–32.4) months, suggesting that the use of nab-PTX instead of PTX was beneficial in terms of OS and PFS in the Japanese population.

In conventional PTX therapy, peripheral neuropathy is the limiting AE. Analysis of PTX + CBDCA + BEV therapy as first-line treatment for Japanese patients with non-squamous NSCLC showed development of Grade ≥ 3 peripheral neuropathy in 5% of the patients, while that of Grade 1 or 2 in 81% (9). We did not observe Grade ≥ 3 peripheral neuropathy in any patient in this study, and Grade 1 or 2 was reported in 56% of patients in Phase I and in 65% in Phase II, suggesting that nab-PTX reduces the occurrence of peripheral neuropathy.

Immune checkpoint inhibitors targeting programmed cell death protein 1 and programmed death ligand-1, such as atezolizumab, pembrolizumab and nivolumab, significantly improve OS in patients with advanced NSCLC compared with chemotherapy (20–23). Moreover, combination treatment of immune checkpoint inhibitors with standard chemotherapy for advanced NSCLC has demonstrated improved outcomes compared with standard chemotherapy (24–29). The results of the IMpower130 trial showed that the addition of atezolizumab to nab-PTX + CBDCA standard chemotherapy as first-line therapy for advanced non-squamous NSCLC improved the OS (18.6 vs. 13.9 months) and PFS (7.0 vs. 5.5 months) (29). In KEYNOTE-407, which compared the combination of pembrolizumab + CBDCA + PTX with pembrolizumab + CBDCA + nab-PTX, treatment efficacy did not differ significantly, but the frequency of peripheral neuropathy of all grades was 24.9% and that of Grade ≥ 3 was 1.2% in the PTX group, while that of all grades was <15% in the nab-PTX group (27). Considering the adverse event profile, nab-PTX may also be a treatment option in combination chemotherapy with immune checkpoint inhibitors.

The addition of atezolizumab to the combination of chemotherapeutic agents (PTX + CBCA) and BEV in the IMpower150 trial showed improvements in OS (19.2 vs. 14.7 months) and PFS (8.3 vs. 6.8 months) (26), and the use of anti-angiogenic agents, such as BEV, may be beneficial in patients who progress on the combination of immune checkpoint inhibitors and chemotherapy (30–32). A successfully completed Phase I clinical trial with Japanese patients using the combination of nivolumab and PTX + CBDCA + BEV showed a favorable toxicity profile and encouraging tumor response (33). Since nab-PTX has advantages over conventional PTX in terms of toxicity and response rate, a positive outcome is expected with combination therapy of nab-PTX + CBDCA + BEV with immune checkpoint inhibitors. Therefore, future development of combination therapies with immune checkpoint inhibitors and BEV must be considered.

The main limitation of this study was its single-arm, open-label design. Moreover, the inclusion of only Japanese patients limits the generalization of the results. Further international studies including a larger number of patients are needed.

To our knowledge, this is the first study to evaluate the safety and response rate of the nab-PTX + CBDCA + BEV combination in a Japanese population. The overall response rate based on central judgment was 56.5% (90% CI: 44.5–68.5), and the primary endpoint was achieved. Therefore, nab-PTX + CBDCA + BEV can be considered favorable and is well-tolerated in Japanese patients as first-line treatment for advanced non-squamous NSCLC.
